# Pediatric massage therapy for treatment of tic disorders in children: A systematic review and meta-analysis of randomized controlled trials

**DOI:** 10.1097/MD.0000000000037568

**Published:** 2024-03-22

**Authors:** Jiaqi Wu, Fushuang Yang, Zhongtian Wang, Lie Wang, Tong Tian, Zhilong Xue, Liping Sun

**Affiliations:** a College of Chinese Medicine, Changchun University of Chinese Medicine, Changchun, Jilin, China; b Affiliated Hospital of Changchun University of Chinese Medicine, Changchun, Jilin, China.

**Keywords:** complementary therapy, external treatment of TCM, meta-analysis, pediatric massage therapy, systematic review, tic disorders

## Abstract

**Background:**

Tic disorder is a common neurodevelopmental disorder in childhood, characterized primarily by motor or vocal tics. However, there is no systematic evaluation of pediatric massage therapy for children with Tic disorder. This study aims to evaluate the effectiveness and safety of massage therapy for children with tic disorder through a comprehensive meta-analysis and systematic review.

**Methods:**

We systematically searched relevant randomized controlled trials from various databases such as CBM, CNKI, VIP, Wanfang database, PubMed, Embase, Web of Science, Cochrane Library, and SINOMED, published up to October 2023. To collect randomized controlled trials on pediatric massage therapy or in combination with other therapies for the treatment of tic disorders in children. The risk of bias in the included articles was assessed using the Cochrane guideline. Meta-analyses were performed using Review Manager 5.4, and publication bias was evaluated by using Begg test and Egger test in Stata SE software.

**Results:**

This meta-analysis included 19 randomized controlled trials with 1423 patients. Pediatric massage therapy alone or in combination with conventional medication demonstrated a significant increase in clinical effectiveness rates [risk ratios = 1.15, 95% confidence interval [CI] (1.10, 1.20), Z = 6.54, *P* < .001], and reduced Yale Global Tie Severity Scale scores [standardized mean difference = −0.85, 95% CI (−1.50, −0.19), Z = 2.54, *P* = .01] and traditional Chinese medicine syndrome scores [standardized mean difference = −1.35, 95%CI (−2.08, −0.63), Z = 3.66, *P* = .0002]. In terms of adverse reactions, there was no statistical difference between the experimental and control groups [risk ratios = 0.26, 95% CI (0.14, 0.49), Z = 4.25, *P* < .001]. The Begg test and Egger test results indicated no publication bias.

**Conclusion:**

Evidence suggests that pediatric massage therapy is effective in improving tic disorders in children.

## 1. Introduction

Tic disorder (TD) is a common neurodevelopmental disorder in childhood, characterized primarily by motor or vocal tics.^[1]^ According to DSM-5,^[2]^ TD can be classified into provisional tic disorder, chronic motor or vocal tic disorder, and Tourette syndrome. According to a study by the Centers for Disease Control and Prevention, based on data from the 2007 National Survey of Children’s Health, the estimated prevalence of a lifetime diagnosis of Tourette syndrome by parent report was 3.0 per 1000.^[3]^ In China, the prevalence of TD is estimated at approximately 6.1%, with higher rates among boys than girls.^[4]^ TD typically manifests in childhood with varying severity over time, and most children experience improvements in symptoms in late adolescence or early adulthood.^[5]^ In children, tics can significantly impact school life and have lasting effects on emotional, social, and physical well-being until adulthood.^[6]^

In Western medicine, colistin and guanfacine are used to treat TD, but these drugs often come with side effects such as drowsiness, lightheadedness, tiredness, irritability, and hypotension.^[7]^ Cognitive-behavioral intervention for tics is effective in reducing tic severity and improving the quality of life in Chinese children and adolescents with TD, though finding a trained doctor can be challenging.^[8]^ Additionally, parents of children with special medical needs use a complementary or alternative method for treatment.^[9]^ Therefore, it is essential to identify an effective complementary or alternative approach to address TD.

Massage therapy is one of the widely used alternative therapies. Pediatric massage therapy, rooted in Chinese medicine with modern scientific principles, employs specific techniques to stimulate body acupuncture points and meridians to achieve therapeutic purposes. It is a noninvasive treatment, well-accepted by children, and effective in clinical applications for conditions such as cough, recurrent respiratory infections, acute diarrhea, congenital myxomatosis, and anorexia.^[10–15]^ From the perspective of traditional Chinese medicine (TCM), the pathogenesis of TD in children is hyperactivity of Yang and disharmony of Yin and Yang. Massage aims to restore yin and yang balance. It may be achieved through the stimulation of pressure receptors, resulting in increased vagal activity and reduced cortisol levels.^[16]^ However, there are some limitations, as results vary from doctor to doctor due to different practices. Previous studies seem to indicate that massage therapy, in combination with medication or alone, is effective in improving neurobehavioral disorders in children and has fewer side effects compared to medication.^[17]^ In recent years, a growing number of researchers have explored pediatric massage to treat children with TD, but there is no systematic evaluation of pediatric massage therapy for children with TD. Therefore, we conducted a comprehensive systematic review and meta-analysis based on existing evidence to summarize the evidence of the efficacy and safety of massage therapy for TD in children. This meta-analysis will provide an evidence-based medical basis for pediatric massage therapy in the treatment of childhood tic disorders, bring guidance for clinicians, inspire ideas for researchers, and benefit more patients.

## 2. Materials and methods

This study was performed following the PRISMA 2020 (The Preferred Reporting Items for Systematic Reviews and Meta-Analyses 2020) guideline^[18]^ and registered on PROSPERO (Registration ID: CRD42023396999).This study was based on a statistical analysis of the published literature, it was not necessary to review it by an ethics committee or an institutional board.

### 2.1. Inclusion and exclusion criteria

Inclusion criteria

1. Participants: patients diagnosed with TD, below 18 years, regardless of gender, duration of illness, or source of the case;
2. Study type: randomized controlled trial;
3. Interventions: The treatment group was treated with massage therapy alone or massage combined with conventional medication. The control group was treated with traditional Chinese medicine or Western medicine;
4. Outcome Measures: the primary outcome indicator is total effective rate, whereas secondary outcome indicators include Yale Global Tie Severity Scale (YGTSS) scores, TCM syndrome scores, and adverse effects.

Exclusion criteria

1. Reviews, case reports, nonclinical studies, animal experiments, etc.
2. No clear outcome metrics and efficacy evaluation criteria;
3. Inconsistency of interventions;
4. Full text unavailable.

### 2.2. Retrieval method

A comprehensive search of scientific databases was conducted to identify relevant articles, with no language restrictions. The search period is from the inception of the databases to October, 2023. Both subject words (MeSH) and free words were used for the search. The subject terms included “Tic Disorders” [Mesh] and “Massage” [Mesh]; the keywords were: “Tic Disorder” OR “Chronic Motor or Vocal Tic Disorder” OR “Tic Disorder, Chronic Motor or Vocal” OR “Motor or Vocal Tic Disorder, Chronic” OR “Transient Tic Disorder” OR “Transient Tic Disorders” OR “Childhood Tic Disorders” OR “Childhood Tic Disorder” OR “Tic Disorder, Childhood” OR “Tic Disorders, Childhood” OR “Motor Tic Disorders” OR “Motor Tic Disorder” OR “Tic Disorder, Motor” OR “Tic Disorders, Motor” in combination with “Massage” OR “Zone Therapy” OR “Therapies, Zone” OR “Zone Therapies” OR “Therapy, Zone” OR “Massage Therapy” OR “Massage Therapies” OR “Therapies, Massage” OR “Therapy, Massage.” The search strategy is shown in Annex S1, Supplemental Digital Content, http://links.lww.com/MD/L946.

### 2.3. Literature screening and data extraction.

Two investigators (JQ Wu and FS Yang) independently screened the literature. The retrieved studies were imported into EndNote X9 to remove duplicate records. Then, the titles and abstracts were checked to filter out irrelevant literature. The full texts of the remaining studies were downloaded and comprehensively read to select eligible studies. In case of any disagreements, a third reviewer (LP Sun) was consulted to discuss and resolve the discrepancies. Relevant data such as first author, country, study design, year of publication, sample size, age of subjects, gender, duration of disease, duration of treatment, method of intervention, duration of follow-up, and outcome indicators were extracted from the included studies.

### 2.4. Risk of bias assessment

Two independent reviewers (JQ Wu and FS Yang) assessed the methodological quality and risk of bias in the included randomized controlled trials (RCTs) using the Cochrane risk of bias tool. The quality assessment involves the following domains: random sequence generation, allocation concealment, blinding methods, incomplete outcome data, selective outcome reporting, and other bias sources. The risk of bias was classified as “low,” “high,” or “unclear.”^[19]^ The kappa value of the reviewers who extracted the data was analyzed using spss 27.0 and the kappa value was 0.869, suggesting good consistency.

### 2.5. Statistical analysis

This meta-analysis was performed using the RM 5.4 software. Dichotomous variables were expressed as the odds ratio or risk ratio (RR) with 95% confidence interval (CI), whereas continuous variables were displayed as the mean difference or standardized mean difference (SMD) with 95% CI. Statistically significant differences were considered when the *P*-value was <.05. The heterogeneity of the study results was determined based on the Chi² test, and the values of I² > 25%, 50%, and 75%, indicated low, moderate, and high heterogeneity, respectively. If I² is <50%, the heterogeneity can be ignored. In cases where studies exhibited statistical homogeneity (I^2^ < 50%, *P* > .1), meta-analysis was performed using a fixed effects model. If there was significant heterogeneity (I^2^ ≥ 50%, *P* < .1), meta-analysis was performed using the random effects model, and the source of heterogeneity was further examined by sensitivity analysis, subgroup analysis, or similar techniques.

### 2.6. Publication bias

When the number of included RCTs for an outcome measure was ≥10, Begg test, Egger test and funnel plots were employed to assess the potential publication bias of RCTs.^[20,21]^

## 3. Results

### 3.1. Process and results of literature screening

**Figure 1.**
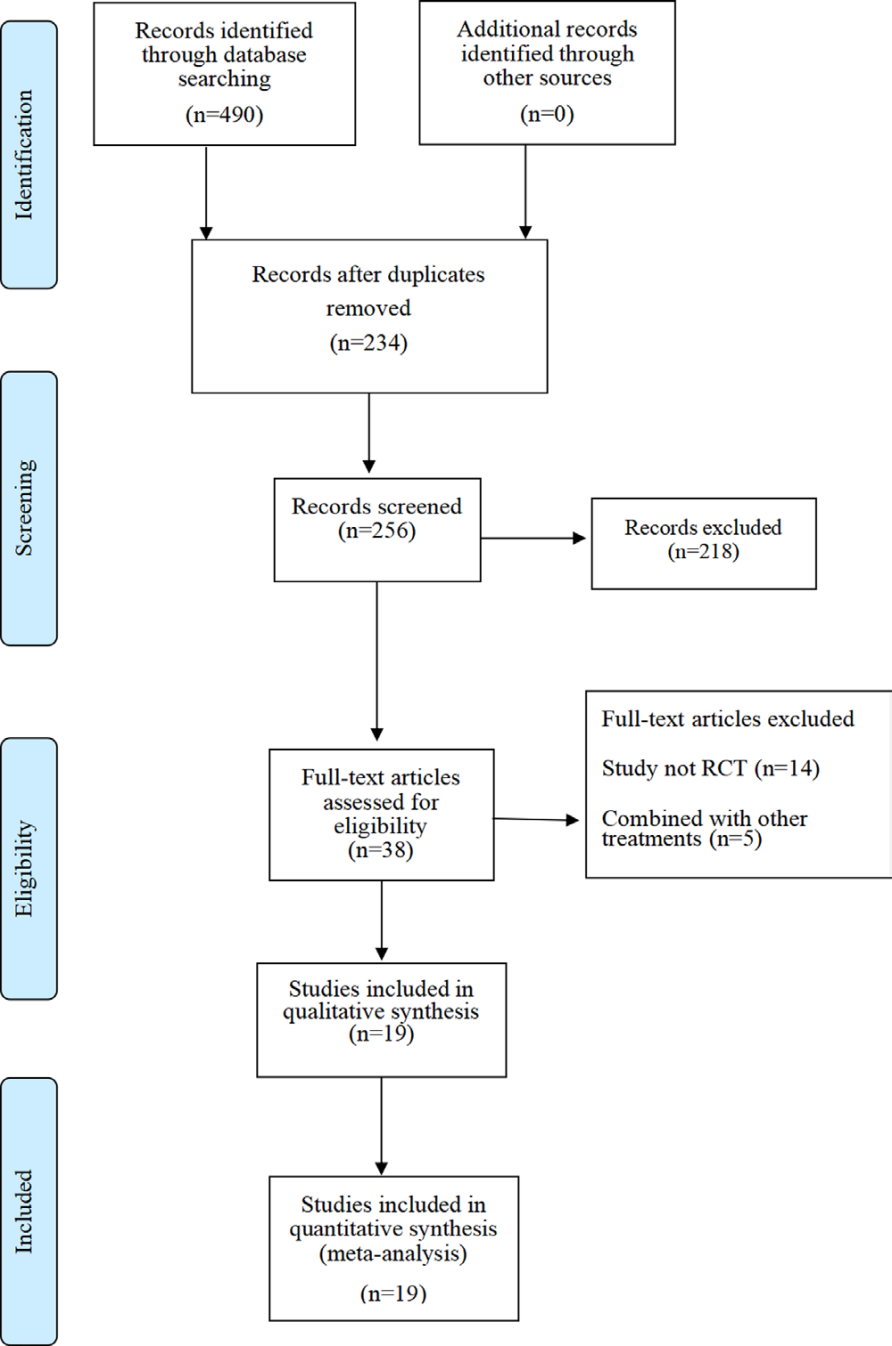
The literature screening process.

Initially, 704 studies were retrieved from PubMed (n = 7), Embase (n = 153), Cochrane Library (n = 195), Web of Science (n = 129), CNKI (n = 57), Wanfang (n = 74), VIP (n = 30), and CBM (n = 59). After literature screening, 19 RCTs were included in this meta-analysis, including CNKI (n = 16), Wanfang (n = 2), VIP (n = 1). The literature screening process is delineated in Figure 1.

### 3.2. Basic characteristics and quality evaluation of the included RCTs.

A total of 19 RCTs^[22–40]^ published in Chinese were included in the analysis. There were 1423 participants, with 711 individuals in the experimental group and 712 individuals in the control group. All the studies were conducted in China. The control group received conventional drug therapy, and the experimental group was given massage therapy alone or combined with conventional drug therapy. The duration of treatment ranged from 4 to 12 weeks. The detailed features of the included studies are shown in Table 1.

**Table 1:** Basic information of the included literature.

Number	Year	The first author	Study type	Country	Intervention		Sample size (E/C)	Outcome measures	Period of treatment	Diagnosis of diseases
					Experimental group	Control group				
1	2022	Yang L	RCT	China	Combined pediatric tuina therapy in the control group	Oral aripiprazole	43/42	①③	12 weeks	TS
2	2017	Mi JQ	RCT	China	Pediatric tuina Therapy	Oral jingling oral liquid	60/60	①	3 months	PTD
3	2018	Zhang LL	RCT	China	Pediatric tuina Therapy	Treatment with thiopride hydrochloride tablets	40/40	①②③	10 weeks	TS
4	2020	Yang XY	RCT	China	In the control group treatment based on the combination of pediatric tuina therapy	Oral haloperidol tablets	45/45	②③④	4 weeks	TD
5	2017	Song HL	RCT	China	Combined pediatric tuina therapy in the control group	Oral thiopride hydrochloride	56/56	①②	4 weeks	TD
6	2019	He T	RCT	China	Liao tuina manipulations	Oral thiopride hydrochloride	32/32	①②④	12 weeks	PTD
7	2011	Zhao P	RCT	China	Pediatric tuina therapy	Oral herbal medicine	20/20	①②	6 weeks	CTD
8	2019	Ji YY	RCT	China	Pediatric tuina therapy	Oral herbal medicine	32/31	①②	4 weeks	TS
9	2020	Shen F	RCT	China	Pediatric tuina therapy	Oral treatment with proprietary Chinese medicines	30/30	①②③	20 days	TD
10	2016	Wu XZ	RCT	China	Combined pediatric tuina therapy in the control group	Oral treatment with Chinese medicine	52/52	①	1 months	TS
11	2022	Huang J	RCT	China	Visceral acupoint massage therapy	Coladine transdermal patch topical treatment	34/36	①②④	28 days	TD
12	2018	Niu LL	RCT	China	In the control group with tuina therapy	Oral herbal tonics	34/34	①④	60 days	TS
13	2018	Du CY	RCT	China	Visceral acupoint massage therapy	Oral haloperidol	42/43	①②④	30 days	TS
14	2016	Du YR	RCT	China	Oral Chinese medicine combined with pediatric massage therapy	Oral thiopride hydrochloride	36/36	①④	60 days	TS
15	2015	Wei J	RCT	China	Oral Chinese medicine combined with pediatric massage therapy	Oral thiopride hydrochloride	30/30	①④	12 weeks	TS
16	2019	Tian X	RCT	China	Pediatric massage combined with oral Chinese medicine	Coladine transdermal patch topical treatment combined with oral herbal medicine	30/30	①②③④	12 weeks	TS comorbidity ADHD
17	2018	Zhang XJ	RCT	China	Combined pediatric tuina therapy in the control group	Oral herbal tonics	30/30	①②③	3 months	TS
18	2020	Shen HY	RCT	China	Combined pediatric tuina therapy in the control group	Oral herbal tonics	35/35	①②③	4 weeks	TD

ADHD = attention deficit hyperactivity disorder, CTD = chronic motor or vocal tic disorder, E/C = experimental group/control group, PTD = provisional tic disorder, TD = tic disorder, TS = Tourette syndrome, TTD = transient tic disorder.

① Clinical effective rates; ② YGTSS scores; ③ TCM syndrome scores; ④ adverse reaction.

**Figure 2.**
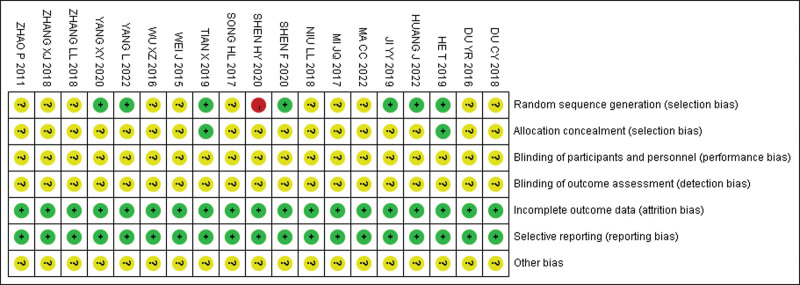
The plot for risk of bias.

Regarding the potential for selection bias, 7 studies^[22,25,27,29,30,32,37]^ employed the random number table method and were rated as having a low risk of bias. One study^[39]^ assigned participants randomly based on the order of visit and was rated as having a high risk of selection bias. The remaining studies did not report the method to generate random sequences, thus categorized as an unclear risk. For allocation concealment bias, 2 studies^[27,37]^ used the sealed envelope bag and were rated as having a low risk. The remaining studies did not report allocation concealment, resulting in an unclear risk. All studies did not report performance bias and detection bias, leading to an unclear risk. For attrition bias, all studies reported prespecified endpoints and were rated as having a low risk. For reporting bias, all studies reported prespecified endpoints and were rated as having a low risk. As for other potential biases, all studies did not provide relevant information, resulting in an unclear risk. The plot for risk of bias is shown in Figure 2.

### 3.3. Meta-analysis results

The meta-analysis results are shown in Table 2.

**Table 2:** The summary of the current meta-analysis results.

Outcome or subgroup	No. of Studies	Participants	Statistical method	Effect size	*P*
Clinical effective rates	18	1333	RR (fixed), 95% CI	1.15 [1.10, 1.20]	<.00001*
			OR (fixed), 95% CI	3.20 [2.24, 4.57]	<.00001*
			RD (fixed), 95% CI	0.12 [0.09, 0.16]	<.00001*
YGTSS scores	12	854	SMD (random), 95% CI	−0.85 [−1.50, −0.19]	.01*
			MD (random), 95% CI	−2.42 [−3.97, −0.88]	.002*
TCM syndrome scores	8	566	SMD (random), 95% CI	−1.35 [−2.08, −0.63]	.0002*
			MD (random), 95% CI	−2.34 [−3.22, −1.45]	<.00001*
Adverse reaction	7	501	RR (fixed), 95% CI	0.26 [0.14, 0.49]	<.0001*
			OR (fixed), 95% CI	0.25 [0.13, 0.47]	<.0001*
			RD (fixed), 95% CI	−0.06 [−0.08, −0.03]	<.00001*

MD = mean difference, OR = odds ratio, RD = risk difference, RR = relative ratio, SMD = standardized mean difference, TCM **=** traditional Chinese medicine, YGTSS **=** Yale Global Tic Severity Scale.

* Favors treatment group with statistical significance.

#### 3.3.1. Clinical effective rates.

**Figure 3.**
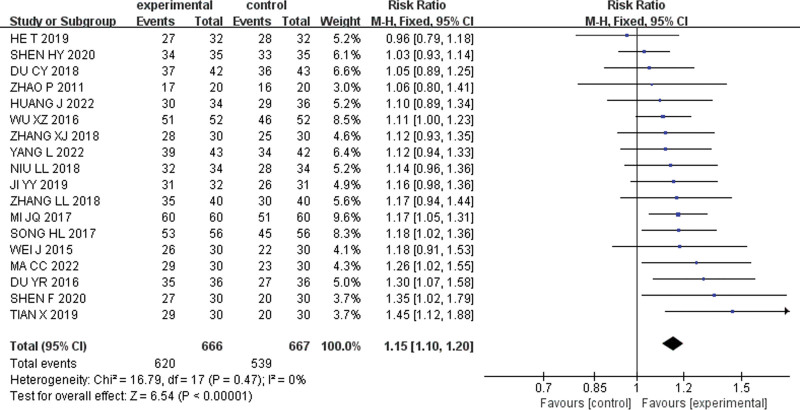
Forest plot for clinical effective rates.

Among the selected literature, 18 articles^[22–24,26–40]^ reported clinical effectiveness rates with a total of 1333 cases, including 666 cases in the experimental group and 667 cases in the control group. No significant heterogeneity was detected (*P* = .48, I^2^ = 0%), and thus a fixed effect model was chosen to combine the effect sizes [RR = 1.15, 95% CI (1.10, 1.20), Z = 6.54, *P* < .001]. According to the forest plot, the diamond weights did not intersect with the null line and were located at the right of the null line, indicating that the clinical effectiveness rate of the experimental group was significantly higher than that of the control group. The results of the meta-analysis of clinical effective rates are shown in Figure 3.

#### 3.3.2. YGTSS scores.

**Figure 4.**
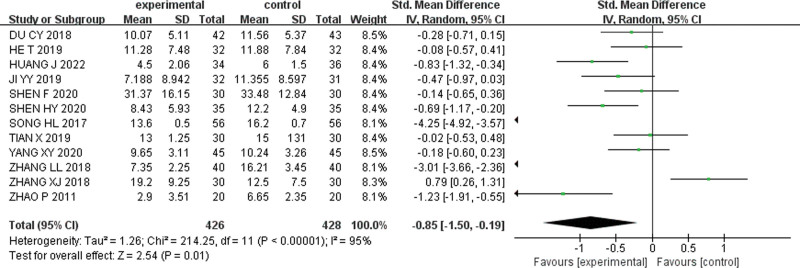
Forest plot for YGTSS score. YGTSS = Yale Global Tie Severity Scale.

Twelve articles^[24–30,32,34,37–39]^ reported YGTSS scores, with 426 cases in the experimental group and 428 cases in the control group. The heterogeneity across these studies was high (I²= 95%). Therefore, the random effect model was used to pool data [SMD = −0.85, 95% CI (−1.50, −0.19), Z = 2.54, *P* = .01]. The analysis showed that the YGTSS score in the experimental group was significantly lower than that in the control group. The forest plot for YGTSS score is presented in Figure 4.

Given the high level of heterogeneity, a sensitivity analysis was performed to investigate potential sources of heterogeneity. These studies were excluded one by one and the results are summarized in Table 3.

**Table 3:** Results of the study after one-by-one exclusion.

Researches	I^2^	*P*-value	SMD 95% CI
Zhang LL 2018	94%	.04	−0.65 [−1.26, −0.04]
Yang XY 2020	95%	.01	−0.91 [−1.64, −0.18]
Song HL 2017	90%	.03	−0.54 [−1.01, −0.06]
He T 2019	95%	.01	−0.92 [−1.63, −0.21]
Zhao P 2011	95%	.02	−0.81 [−1.51, −0.12]
Ji YY 2019	95%	.02	−0.88 [−1.60, −0.17]
Shen F 2020	95%	.01	−0.85 [−1.57, −0.13]
Huang J 2022	95%	.02	−1.50 [−2.61, −0.39]
Du CY 2018	95%	.02	−0.90 [−1.63, −0.17]
Tian X 2019	95%	.01	−0.92 [−1.63, −0.22]
Zhang XJ 2018	95%	.003	−0.99 [−1.66, −0.33]
Shen HY 2020	95%	.02	−0.86 [−1.59, −0.14]

**Figure 5.**
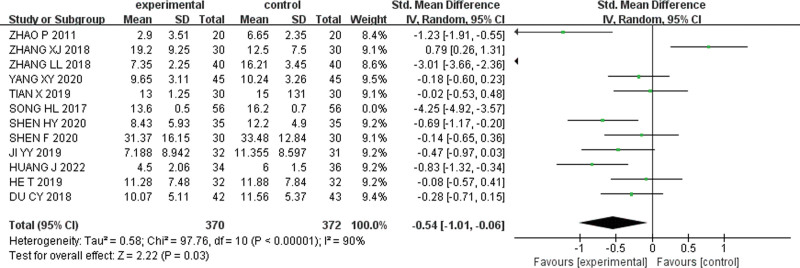
Forest plot excluding Song HL’s study.

When the study by Song HL were excluded, the remaining studies exhibited reduced heterogeneity (I^2^ = 90%). The meta-analysis was performed again using a random-effects model, and the results showed [SMD = −0.54, 95% CI (−1.01, −0.06), Z = 2.22, *P* = .03] (Fig. 5).

**Figure 6.**
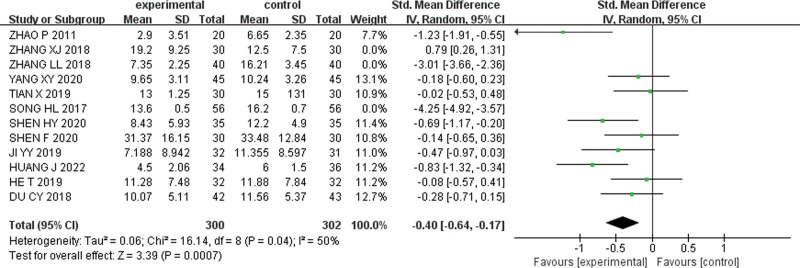
Forest plot after excluding 3 studies.

Further exclusion of 3 articles by Zhang L.L, Zhang X.J, Song HL, caused even lower heterogeneity (I^2^ = 50%), and the random effect model was used for meta-analysis [SMD = −0.40, 95% CI (−0.64, −0.17), Z = 3.39, *P* = .0007]. The results of meta-analysis are shown in Figure 6. As shown in the forest plot, the weighted rhombus did not intersect with the null line and was located to the left of the null line, suggesting that the overall efficacy rate of pediatric massage alone or in combination with conventional drug medication was higher than that of conventional drug medication alone in treating children with TD.

#### 3.3.3. TCM syndrome scores.

**Figure 7.**
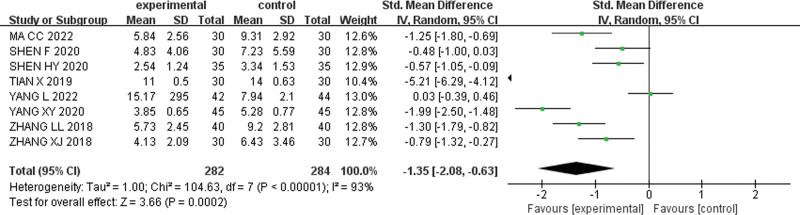
Forest plot for TCM syndrome score. TCM = traditional Chinese medicine.

Eight studies^[22,24,25,30,37–40]^ reported TCM evidence points, with 282 cases in the experimental group and 284 cases in the control group. The analysis demonstrated high heterogeneity (I² = 93%), and thus the random effect model was chosen to combine the effect sizes [SMD = −1.35, 95% CI (−2.08, −0.63), Z = 3.66, *P* = .0002]. Moreover, the TCM syndrome score in the experimental group were significantly lower than that in the control group. The forest plot for TCM syndrome score is shown in Figure 7.

Furthermore, given the high heterogeneity in this outcome indicator, a sensitivity analysis was conducted to explore potential sources of heterogeneity. The included studies were excluded one by one, and the results are shown in Table 4.

**Table 4:** Results of TCM syndrome scores after one-by-one exclusion.

Researches	I^2^	*P*-value	SMD 95% CI
Zhang XJ 2018	94%	.0007	−1.45 [−2.28, −0.61]
Zhang LL 2018	94%	.001	−1.37 [−2.21, −0.53]
Tian X 2019	87%	.0006	−0.90 [−1.41, −0.39]
Shen F 2020	94%	.0004	−1.49 [−2.31, −0.67]
Shen HY 2020	94%	.0005	−1.48 [−2.32, −0.64]
Yang XY 2020	93%	.001	−1.26 [−2.02, −0.49]
Ma CC 2022	94%	.001	−1.38 [−2.20, −0.55]
Yang L 2022	92%	<.0001	−1.55 [−2.30, −0.80]

**Figure 8.**
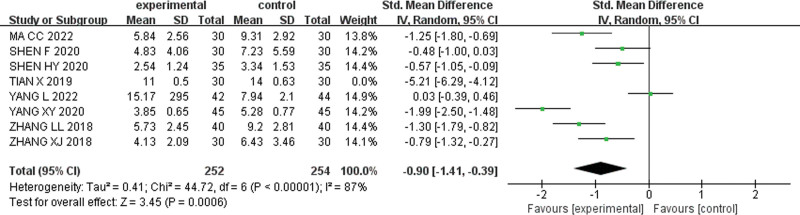
Forest plot excluding Tian X’s study.

**Figure 9.**
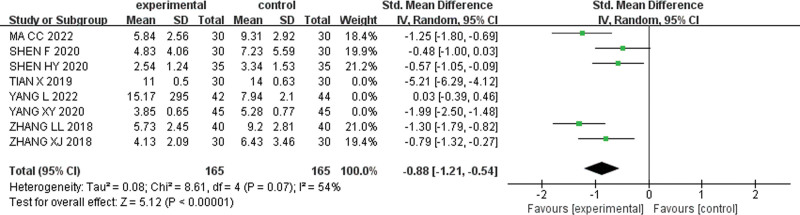
Forest plot after excluding 3 studies.

When the study of Tian X was excluded, the heterogeneity of the remaining studies was significantly reduced (I^2^ = 87%). The forest plot analysis using a random-effects model and combined sizes demonstrated [SMD = −0.90, 95% CI (−1.41, −0.39), Z = 3.45, *P* = .0006] (Fig. 8). Moreover, excluding 3 studies by Yang L, Tian X, and Yang XY, resulted in even lower heterogeneity (I^2^ = 54%), and the random effect model was chosen to combine the effect sizes [SMD = −0.88,95% CI (−1.21, −0.54),Z = 5.12, *P* < .001].The results are shown in Figure 9. As shown in the forest plot, the weighted rhombus did not intersect with the null line and was located to the left of the null line, suggesting that pediatric massage alone or in combination with conventional drug medication was superior to medication alone in improving the TCM syndrome scores of children with TD.

#### 3.3.4. Adverse reactions.

**Figure 10.**
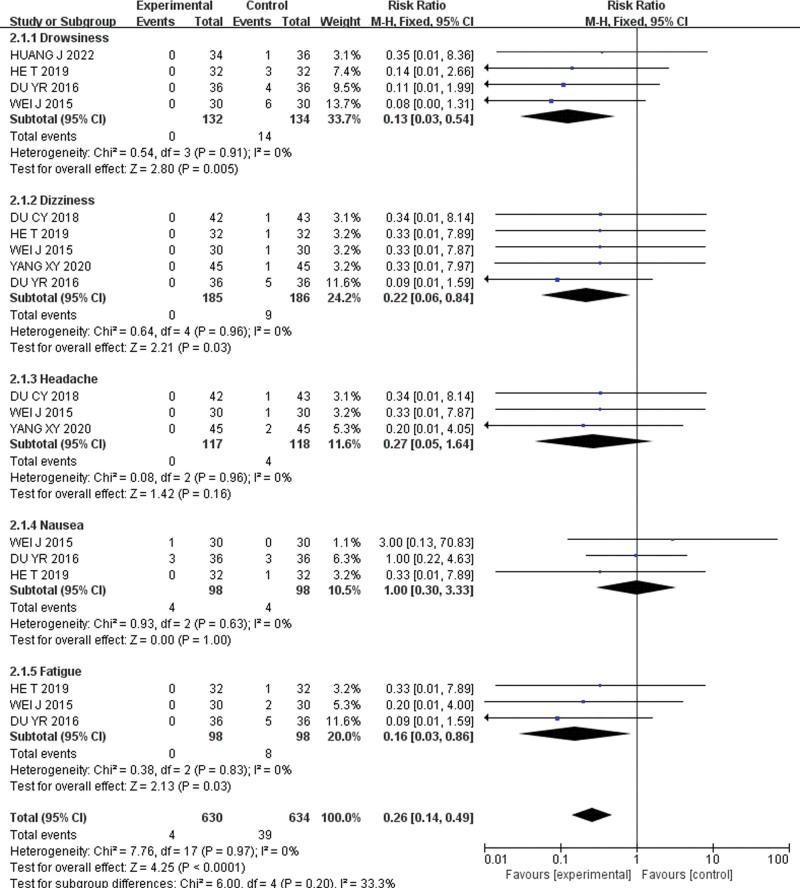
Forest plot for adverse reactions.

Seven studies^[25,27,32,34–37]^ reported adverse reactions, such as drowsiness, dizziness, headache, nausea, and malaise. Subgroup analyses were performed for adverse reactions with high incidence rates. Meta-analysis results are shown in Figure 10. Pediatric acupressure was found to reduce the overall incidence of adverse reactions [RR = 0.26, 95% CI (0.14, 0.49), *P* < .001].

Subgroup analyses further discovered that pediatric acupressure alone or in combination, effectively reduced drowsiness [RR = 0.13, 95% CI (0.03, 0.54), *P* = .005, I^2^ = 0%], dizziness [RR = 0.22, 95% CI (0.06, 0.84), *P* = .03, I^2^ = 0%], headache [RR = 0.27, 95% CI (0.05, 1.64), *P* = .16, I^2^ = 0%], nausea [RR = 1.00, 95% CI (0.30, 3.33), *P* = 1.00, I^2^ = 0%], and fatigue [RR = 0.16, 95% CI (0.03, 0.86), *P* = .03, I^2^ = 0%].

### 3.4. Publication bias

**Figure 11.**
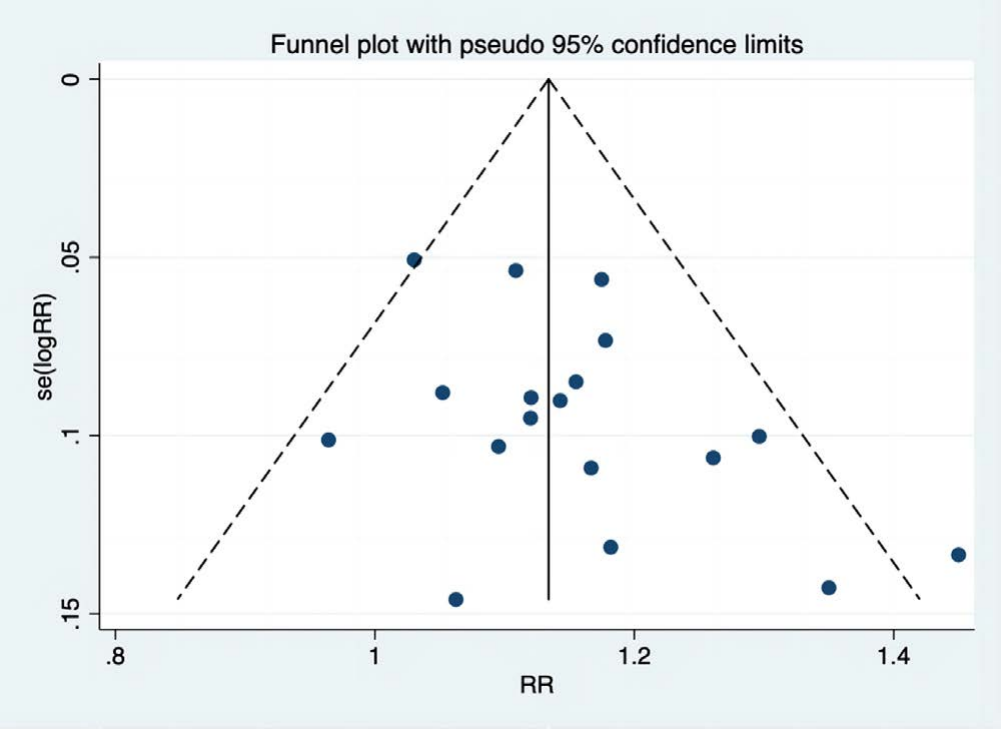
Funnel plot for clinical effective rates.

**Figure 12.**
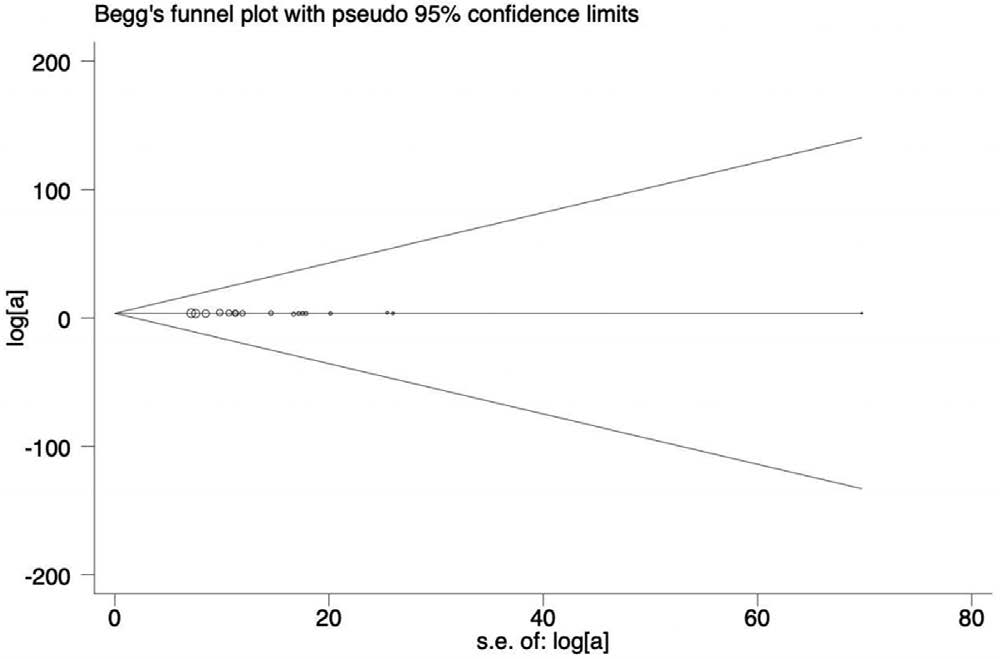
The result of the Begg test.

**Figure 13.**
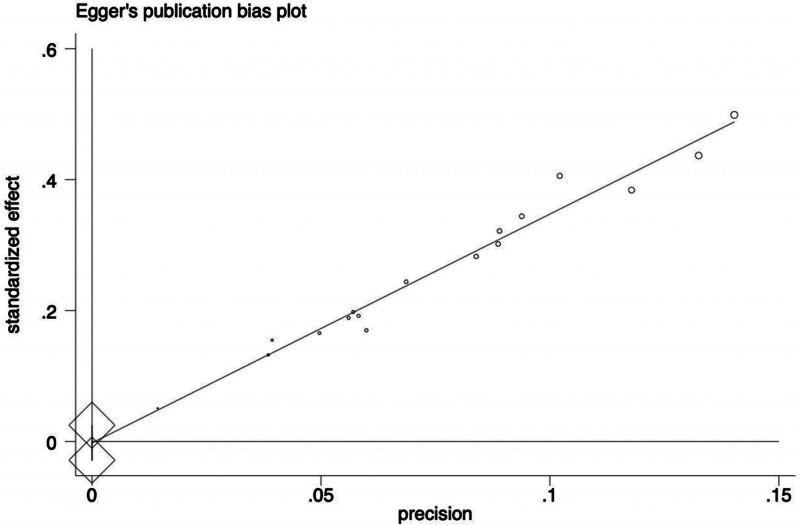
The result of the Egger test.

Publication bias analysis was performed for the primary outcome indicator of clinical efficacy. As indicated in Figure 11, the funnel plot was symmetrical. Begg test (*P* = .12) and Egger test (*P* = .855) were performed and showed no evidence of bias detected, as shown in Figures 12 and 13, respectively.

### 3.5. Sensitivity analysis

In our meta-analysis, the included RCTs were generally homogeneous. The primary outcome indicator was clinical efficacy, while YGTSS scores, TCM syndrome scores and adverse effects were classified as secondary outcome indicators. According to the meta-analysis data, the heterogeneity for YGTSS scores and TCM syndrome scores was high, prompting to carry out sensitivity analysis to investigate the source of heterogeneity. Furthermore, after excluding 3 articles by Zhang L.L, Zhang X.J, and Song HL, the remaining studies exhibited significantly reduced heterogeneity (I^2^ = 50%). In terms of TCM syndrome scores, when the 3 articles by Yang L, Tian X, and Yang XY were excluded, the heterogeneity of the remaining studies decreased (I^2^ = 54%).

Similar results were obtained when subgroup and sensitivity analyses were performed based on the disease diagnosis [RR = 1.15 95% CI (1.11, 1.20), *P* < .001], treatment modality [RR = 1.15, 95% CI (1.10, 1.20), *P* < .001], or sample size (≥60 per group of participants). The results remained consistent and robust [RR = 1.15, 95% CI (1.07, 1.24), *P* = .0002]. Moreover, sensitivity and subgroup analyses showed similar results for YGTSS scores, TCM evidence scores, and adverse reactions.

## 4. Discussion

Pediatric massage is a widely physical therapy for treating TD in children. However, its effectiveness and safety have not been systematically evaluated. To the best of our knowledge, this is the first study to summarize the current evidence on the use of massage for the treatment of TD. This meta-analysis of 19 RCTs indicated that pediatric massage therapy alone or in combination with conventional drug medication was superior to drug therapy alone in ameliorating the symptoms of pediatric TD patients. Furthermore, it exhibited a synergistic effect on the clinical outcome indicators of TD patients. The results of this evidence-based study clearly indicated that pediatric massage therapy could improve several crucial outcome indicators of TD, such as improving clinical effectiveness, and reducing the YGTSS score and TCM syndrome scores. These results strongly suggest that massage therapy can be beneficial for individuals with TD.

It is worth noting that there have been relatively fewer studies on massage therapy for TD. Chen SC et al^[15]^ conducted a meta-analysis of 4 studies, which indicated that massage therapy was beneficial in treating attention deficit hyperactivity disorder in children and adolescents. Both TD and attention deficit hyperactivity disorder are childhood neurodevelopmental disorders,^[41]^ which implies that our findings are similar to the previous study.

Pediatric massage is performed based on the meridian theory, in which the manipulation, strength, and direction of specific acupuncture points can produce diverse therapeutic effects for various diseases. According to traditional Chinese medicine theory, children have predominantly Yang-based constitutions, characterized by excess yang energy and a relative deficiency of essence, blood, and fluid. This imbalance is thought to contribute to the onset of TD. Pediatric massage can strengthen the spleen, soften the liver, and nourish the yin to calm the wind. Regarding the biological mechanism of massage, some studies have found that the application of massage techniques leads to cyclic dynamic changes in tissue pressure. This, in turn, induces changes in osmolality, osmotic factor, and the apparent viscosity of blood in capillaries, promoting better blood circulation and substance exchange.

This meta-analysis exhibits several limitations. First, the relatively small sample size could potentially impact the accuracy of the study results. Since all the included studies were conducted in China, the results might not be applied to different populations. Second, a majority of the included studies did not provide explicit details about their randomization, hidden allocation, or blinding method, which may undermine the reliability of the evidence to a certain extent. Third, there are multiple diagnostic criteria for TD, and the 19 papers included in this study have employed various textbooks, guidelines, and other diagnostic criteria. The lack of standardization in diagnostic criteria introduces heterogeneity in the systematic research, potentially affecting the results.

Large-scale, multicenter studies are desired to validate the results of this meta-analysis. Furthermore, extending the follow-up period to explore the long-term effects of pediatric massage therapy and its potential mechanism of action could offer a more comprehensive understanding of its feasibility as a physical therapy for tic disorders.

## 5. Conclusion

In conclusion, this meta-analysis highlights the potential efficacy and safety of pediatric massage therapy for Tic disorders in children. However, it is important to note that that the included studies exhibited overall low methodological quality and had limitations in the control design. Hence, further research with improved methodological designs is crucial to corroborate the efficacy and safety of massage as a treatment for tic disorders in children.

## Author contributions

**Data curation:** Jiaqi Wu, Fushuang Yang.

**Methodology:** Zhongtian Wang, Tong Tian.

**Software:** Jiaqi Wu, Zhilong Xue.

**Supervision:** Lie Wang.

**Writing – original draft:** Jiaqi Wu.

**Writing – review & editing:** Liping Sun.

## Supplementary Material


