# Supplementary material for: Pediatric massage therapy for treatment of tic disorders in children: A systematic review and meta-analysis of randomized controlled trials
Source: Medicine (Baltimore). 2024 Mar 22;103(12):e37568. doi: 10.1097/MD.0000000000037568 (PMC10957010; doi:10.1097/MD.0000000000037568)
Supplement: Supplementary file 1 [file medi-103-e37568-s001.docx]

**Annex I:** Search strategy

**PubMed**

| **Search** | **Query** | **Results** |
| --- | --- | --- |
| 1 | "Tic Disorders"[MeSH Terms] | 5992 |
| 2 | ((((((((((((((Tic Disorders[Title/Abstract]) OR (Tic Disorder[Title/Abstract])) OR ("Chronic Motor or Vocal Tic Disorder"[Title/Abstract])) OR ("Tic Disorder, Chronic Motor or Vocal"[Title/Abstract])) OR ("Motor or Vocal Tic Disorder, Chronic"[Title/Abstract])) OR (Transient Tic Disorder[Title/Abstract])) OR (Transient Tic Disorders[Title/Abstract])) OR (Childhood Tic Disorders[Title/Abstract])) OR (Childhood Tic Disorder[Title/Abstract])) OR (Tic Disorder, Childhood[Title/Abstract])) OR (Tic Disorders, Childhood[Title/Abstract])) OR (Motor Tic Disorders[Title/Abstract])) OR (Motor Tic Disorder[Title/Abstract])) OR (Tic Disorder, Motor[Title/Abstract])) OR (Tic Disorders, Motor[Title/Abstract]) | 1804 |
| 3 | ("Tic Disorders"[MeSH Terms]) OR (((((((((((((((Tic Disorders[Title/Abstract]) OR (Tic Disorder[Title/Abstract])) OR ("Chronic Motor or Vocal Tic Disorder"[Title/Abstract])) OR ("Tic Disorder, Chronic Motor or Vocal"[Title/Abstract])) OR ("Motor or Vocal Tic Disorder, Chronic"[Title/Abstract])) OR (Transient Tic Disorder[Title/Abstract])) OR (Transient Tic Disorders[Title/Abstract])) OR (Childhood Tic Disorders[Title/Abstract])) OR (Childhood Tic Disorder[Title/Abstract])) OR (Tic Disorder, Childhood[Title/Abstract])) OR (Tic Disorders, Childhood[Title/Abstract])) OR (Motor Tic Disorders[Title/Abstract])) OR (Motor Tic Disorder[Title/Abstract])) OR (Tic Disorder, Motor[Title/Abstract])) OR (Tic Disorders, Motor[Title/Abstract])) | 6650 |
| 4 | Massage[MeSH Terms] | 6950 |
| 5 | (((((((Zone Therapy[Title/Abstract]) OR (Therapies, Zone[Title/Abstract])) OR (Zone Therapies[Title/Abstract])) OR (Therapy, Zone[Title/Abstract])) OR (Massage Therapy[Title/Abstract])) OR (Massage Therapies[Title/Abstract])) OR (Therapies, Massage[Title/Abstract])) OR (Therapy, Massage[Title/Abstract]) | 45270 |
| 6 | (Massage[MeSH Terms]) OR ((((((((Zone Therapy[Title/Abstract]) OR (Therapies, Zone[Title/Abstract])) OR (Zone Therapies[Title/Abstract])) OR (Therapy, Zone[Title/Abstract])) OR (Massage Therapy[Title/Abstract])) OR (Massage Therapies[Title/Abstract])) OR (Therapies, Massage[Title/Abstract])) OR (Therapy, Massage[Title/Abstract])) | 51397 |
| 7 | Randomized controlled trials[MeSH Terms] | 167918 |
| 8 | ((randomized controlled trials[Title/Abstract]) OR (randomized[Title/Abstract])) OR (RCT[Title/Abstract]) | 688507 |
| 9 | (Randomized controlled trials[MeSH Terms]) OR (((randomized controlled trials[Title/Abstract]) OR (randomized[Title/Abstract])) OR (RCT[Title/Abstract])) | 774742 |
| 10 | ((("Tic Disorders"[MeSH Terms]) OR (((((((((((((((Tic Disorders[Title/Abstract]) OR (Tic Disorder[Title/Abstract])) OR ("Chronic Motor or Vocal Tic Disorder"[Title/Abstract])) OR ("Tic Disorder, Chronic Motor or Vocal"[Title/Abstract])) OR ("Motor or Vocal Tic Disorder, Chronic"[Title/Abstract])) OR (Transient Tic Disorder[Title/Abstract])) OR (Transient Tic Disorders[Title/Abstract])) OR (Childhood Tic Disorders[Title/Abstract])) OR (Childhood Tic Disorder[Title/Abstract])) OR (Tic Disorder, Childhood[Title/Abstract])) OR (Tic Disorders, Childhood[Title/Abstract])) OR (Motor Tic Disorders[Title/Abstract])) OR (Motor Tic Disorder[Title/Abstract])) OR (Tic Disorder, Motor[Title/Abstract])) OR (Tic Disorders, Motor[Title/Abstract]))) AND ((Massage[MeSH Terms]) OR ((((((((Zone Therapy[Title/Abstract]) OR (Therapies, Zone[Title/Abstract])) OR (Zone Therapies[Title/Abstract])) OR (Therapy, Zone[Title/Abstract])) OR (Massage Therapy[Title/Abstract])) OR (Massage Therapies[Title/Abstract])) OR (Therapies, Massage[Title/Abstract])) OR (Therapy, Massage[Title/Abstract])))) AND ((Randomized controlled trials[MeSH Terms]) OR (((randomized controlled trials[Title/Abstract]) OR (randomized[Title/Abstract])) OR (RCT[Title/Abstract]))) | 0 |

**Web of Science**

| **Search** | **Query** | **Results** |
| --- | --- | --- |
| 1 | (((((((((((((((TS=(Tic Disorders)) OR TS=(Tic Disorder))) OR TS=(Chronic Motor or Vocal Tic Disorder)) OR TS=(Tic Disorder, Chronic Motor or Vocal)) OR TS=(Motor or Vocal Tic Disorder, Chronic)) OR TS=(Transient Tic Disorder)) OR TS=(Transient Tic Disorders)) OR TS=(Childhood Tic Disorders)) OR TS=(Childhood Tic Disorder)) OR TS=(Tic Disorder, Childhood)) OR TS=(Tic Disorders, Childhood)) OR TS=(Motor Tic Disorders)) OR TS=(Motor Tic Disorder)) OR TS=(Tic Disorder, Motor)) OR TS=(Tic Disorders, Motor) | 395798 |
| 2 | ((((((((TS=(Massage)) OR TS=(Zone Therapy)) OR TS=(Therapies, Zone)) OR TS=(Zone Therapies)) OR TS=(Therapy, Zone)) OR TS=(Massage Therapy)) OR TS=(Massage Therapies)) OR TS=(Therapies, Massage)) OR TS=(Therapy, Massage) | 18419 |
| 3 | (((TS=(Randomized controlled trials)) OR TS=(randomized controlled trials)) OR TS=(randomized)) OR TS=(RCT) | 829687 |
| 4 | #1 AND #2 AND #3 | 83 |

**Embase**

| **Search** | **Query** | **Results** |
| --- | --- | --- |
| 1 | 'tic disorders'/exp | 16318 |
| 2 | 'tic disorders':ab,ti OR 'tic disorder':ab,ti OR 'chronic motor':ab,ti OR 'vocal tic disorder':ab,ti OR 'tic disorder, chronic motor':ab,ti OR vocal:ab,ti OR motor:ab,ti OR 'vocal tic disorder, chronic':ab,ti OR 'transient tic disorder':ab,ti OR 'transient tic disorders':ab,ti OR 'childhood tic disorders':ab,ti OR 'childhood tic disorder':ab,ti OR 'tic disorder, childhood':ab,ti OR 'tic disorders, childhood':ab,ti OR 'motor tic disorders':ab,ti OR 'motor tic disorder':ab,ti OR 'tic disorder, motor':ab,ti OR 'tic disorders, motor':ab,ti | 547106 |
| 3 | #1 OR #2 | 558743 |
| 4 | 'massage'/exp | 18568 |
| 5 | massage:ab,ti OR 'zone therapy':ab,ti OR 'therapies, zone':ab,ti OR 'zone therapies':ab,ti OR 'therapy, zone':ab,ti OR 'massage therapy':ab,ti OR 'massage therapies':ab,ti OR 'therapies, massage':ab,ti OR 'therapy, massage':ab,ti | 16570 |
| 6 | #4 OR #5 | 25123 |
| 7 | 'randomized controlled trials'/exp | 261830 |
| 8 | 'randomized controlled trials':ab,ti OR andomized:ab,ti OR rct:ab,ti | 161777 |
| 9 | #7 OR #8 | 353036 |
| 10 | #3 AND #6 AND #9 | 51 |

**Cochrane Library**

| **Search** | **Query** | **Results** |
| --- | --- | --- |
| 1 | MeSH descriptor: [Tic Disorders] explode all trees | 379 |
| 2 | (Tic Disorders):ti,ab,kw OR (Tic Disorder):ti,ab,kw OR (Chronic Motor or Vocal Tic Disorder):ti,ab,kw OR (Tic Disorder, Chronic Motor or Vocal):ti,ab,kw OR (Motor or Vocal Tic Disorder, Chronic):ti,ab,kw | 53651 |
| 3 | (Transient Tic Disorder):ti,ab,kw OR (Transient Tic Disorders):ti,ab,kw OR (Childhood Tic Disorders):ti,ab,kw OR (Childhood Tic Disorder):ti,ab,kw OR (Tic Disorder, Childhood):ti,ab,kw | 93 |
| 4 | (Tic Disorders, Childhood):ti,ab,kw OR (Motor Tic Disorders):ti,ab,kw OR (Motor Tic Disorder):ti,ab,kw OR (Tic Disorder, Motor):ti,ab,kw OR (Tic Disorders, Motor):ti,ab,kw | 228 |
| 5 | #1 or #2 or #3 or #4 | 537734 |
| 6 | MeSH descriptor: [Massage] explode all trees | 1695 |
| 7 | (Zone Therapy):ti,ab,kw OR (Therapies, Zone):ti,ab,kw OR (Zone Therapies):ti,ab,kw OR (Therapy, Zone):ti,ab,kw OR (Massage Therapy):ti,ab,kw | 6311 |
| 8 | (Massage Therapies):ti,ab,kw OR (Therapies, Massage):ti,ab,kw OR (Therapy, Massage):ti,ab,kw OR (Massage):ti,ab,kw | 7413 |
| 9 | #6 or #7 or #8 | 9811 |
| 10 | MeSH descriptor: [Randomized Controlled Trial] explode all trees | 25733 |
| 11 | (randomized controlled trials):ti,ab,kw OR (randomized):ti,ab,kw OR (RCT):ti,ab,kw | 1265486 |
| 12 | #10 or #11 | 1265486 |
| 13 | #5 and #9 and #12 | 356 |
